# Population-Level Resources and Costs Associated with Low-Risk Prostate Cancer Patients on Active Surveillance

**DOI:** 10.3390/cancers18101580

**Published:** 2026-05-13

**Authors:** Soo Jin Seung, Jane Bayani, Lena Nguyen, Ning Liu, Jodi Gatley, Anisia Wong, Emilie Richard, Sarah L. Barker, Anna Ying-Wah Lee, John M. S. Bartlett, David Berman, Andrew Loblaw, Craig C. Earle, Nicole Mittmann

**Affiliations:** 1 HOPE Research Centre, Sunnybrook Research Institute, Toronto, ON M4N 3M5, Canada; anisia.wong@sri.utoronto.ca (A.W.); emilie.richard@queensu.ca (E.R.); 2 Ontario Institute for Cancer Research, Toronto, ON M5G 0A3, Canada; jbayani@oicr.on.ca (J.B.); sbarker@oicr.on.ca (S.L.B.); ayingwahlee@oicr.on.ca (A.Y.-W.L.); 3 Institute for Clinical Evaluative Sciences, Toronto, ON M4N 3M5, Canada; lena.nguyen@ices.on.ca (L.N.); ning.liu@ices.on.ca (N.L.); jodi.gatley@ices.on.ca (J.G.); craig.earle@ices.on.ca (C.C.E.); 4 Department of Laboratory Medicine & Pathobiology, University of Edinburgh, Edinburgh EH8 9YL, UK; john.bartlett@ed.ac.uk; 5 Department of Pathology & Molecular Medicine, Queen’s University, Kingston, ON K7L 3N6, Canada; bermand@queensu.ca; 6 Sunnybrook Health Sciences Centre, Toronto, ON M4N 3M5, Canada; andrew.loblaw@sunnybrook.ca (A.L.);; 7 Canadian Partnership Against Cancer, Toronto, ON M5H 1J8, Canada

**Keywords:** prostate cancer, low-risk prostate cancer, active surveillance, costs, healthcare resource utilization

## Abstract

Background: This study was conducted to evaluate the population-level healthcare resource utilization (HCRU) and costs for men diagnosed with low-risk prostate cancer (PCa) either on active surveillance (AS) or not on AS, in which AS was defined as receiving no treatment within 1 year of diagnosis and two biopsies. Methods: AS men aged 40 to 105 years, diagnosed with stage I or II PCa, had a prostate-specific antigen (PSA) level < 20 ng/mL, a Gleason score between 5 and 7, and were matched (1:1) with men not receiving AS. The index date is defined as the date 1 year after PCa diagnosis. HCRU and costs were assessed using a macro-based costing methodology and costs standardized to 2023 CAD. Means (SD) and medians (interquartile ranges) per person-year values were reported annually. Results: During the year leading up to the index date, the mean number of HCRU per patient-year (PPY) was significantly lower for the AS cases versus non-AS in terms of cancer clinic visits (1.7 vs. 26.7), hospital outpatient clinic visits (3.6 vs. 4.9), all physician visits (16.3 vs. 17.5), and specialist visits (10.7 vs. 11.6). The mean overall cost PPY was $6100 ± $12,400 for AS cases and $10,400 ± $17,800 for non-AS men (median overall cost PPY= $3500 [IQR: $2100–$5700] vs. $3700 [IQR: $2100–$7200] (*p* = 0.0001, respectively). Conclusions: HCRU and costs calculated for AS and non-AS low-risk PCa men indicate the cost savings potential for AS.

## Simple Summary

This study looked at healthcare use and costs among men with low-risk prostate cancer who either followed active surveillance (AS) or did not. Active surveillance means closely monitoring cancer instead of starting treatment right away. Researchers matched men in both groups and examined their healthcare visits and costs in Ontario. They found that men on active surveillance had fewer healthcare visits, including cancer clinic visits, hospital outpatient visits, and specialist visits. Healthcare costs were also lower for men on active surveillance, averaging about $6050 per year compared with $10,438 per year for men who were not on surveillance. Overall, the findings suggest that active surveillance can reduce healthcare use and costs for men with low-risk prostate cancer, while still allowing their condition to be carefully monitored. This approach may help patients avoid unnecessary treatment and reduce strain on the healthcare system.

## 1. Introduction

In 2024, an estimated 27,900 Canadian men will be diagnosed with prostate cancer (PCa) and 5000 deaths are expected [1]. There are three general PCa risk groups based on test findings such as digital rectal exam (DRE), prostate-specific antigen (PSA), and prostate biopsy: (very) low risk, intermediate risk, and high risk. Very low-risk and low-risk PCa are defined by tumours confined to the prostate, a PSA level of <10 ng/mL, grade group (GG) 1 (equivalent to Gleason Score, GS ≤ 6), fewer than three biopsy cores positive, and cancer detected in ≤50% of those cores [2,3].

For those with very low and low-risk disease, conservative management such as active surveillance (AS) is recommended so that aggressive treatment and related adverse events can be avoided [1,4]. According to guidelines from Cancer Care Ontario (CCO), the AS protocol includes a PSA test every 3 to 6 months, an annual digital rectal exam (DRE), and a confirmatory transrectal ultrasound (TRUS) biopsy with 12–14 cores within 6 to 12 months of AS initiation [5]. Subsequent biopsies are recommended at least every 3 to 5 years, though frequency may vary based on patient-specific factors such as age and risk of progression [5,6]. The intention of AS is to reduce the risk of overtreatment of clinically insignificant PCa, with the option to initiate more invasive interventions if and when the risk level increases [7]. AS has been demonstrated as a safe and feasible option for men with low-grade and favourable-risk intermediate PCa, with 10-year metastasis-free survival at 94.2% and overall survival at 88.7%, and 15-year mortality rates comparable to patients managed with initial definitive intervention [8].

A 2014 cost comparison study modelled outcomes for 12,750 prostate cancer (PCa) patients to evaluate the economic impact of active surveillance (AS) versus immediate treatment from a single health centre [9]. The study estimated a mean first-year cost of $6200 (2012 CAD) per patient on AS. However, as a model-based analysis conducted over a decade ago, its findings may not reflect current costs. In contrast, the present study used an actual cohort of low-risk PCa patients managed with AS, incorporated relevant clinical characteristics such as disease stage, GS, and PSA values, and applied 1:1 matching based on year of PCa diagnosis and propensity scores to identify a comparable non-AS group. Therefore, this allowed for the conduct of study objectives to determine and compare all-cause healthcare resource utilization (HCRU) and costs between AS and non-AS patients.

## 2. Materials and Methods

### 2.1. Study Design

The AS cohort included adult men throughout Ontario diagnosed with stage I or II prostate cancer between 1 January 2010 and 31 December 2015, with a PSA value < 20 ng/mL and a Gleason score of 5–7. Men were classified as initiating AS if they did not receive any definitive treatment (surgery, radiotherapy, or brachytherapy) within one year of diagnosis and had a confirmatory biopsy within two years of the diagnostic biopsy (defined as a second biopsy performed > 60 days after the initial biopsy). The index date was defined as the date 1 year after PCa diagnosis (i.e., if the date of PCa diagnosis was day 1, then the index date was day 366), and the pre-index period was defined as Year 0. The observation window was terminated in the event of death, loss of OHIP eligibility for ≥90 days, any secondary cancer diagnosis in the Ontario Cancer Registry (OCR), and the maximum follow-up date of 31 March 2023—or whichever came first.

In this study, men diagnosed with PCa but not enroled in the prostate cancer AS cohort were matched to the AS cases without replacement and considered as non-AS cases. There was a 1:1 matching on the year of cancer diagnosis and propensity score. The propensity score of a case was calculated based on age, Charlson Co-morbidity Index (CCI), PSA value category (either 0 ng/mL to <10 ng/mL or ≥10 ng/mL to <20 ng/mL), LHIN, rurality, income quintile, and cancer stage.

The AS cases (*N* = 9693) in this study were part of an existing AS dataset from CCO. To confirm their PCa diagnosis, their unique identifier was linked to the OCR and then restricted to men who were diagnosed with PCa between 1 January 2010 and 31 December 2015. The HCRU and costs of all matched AS cases and non-AS cases were followed from pre-index (Year 0) up to six years of follow-up. Costs were standardized to 2023 Canadian dollars.

**Figure 1:**
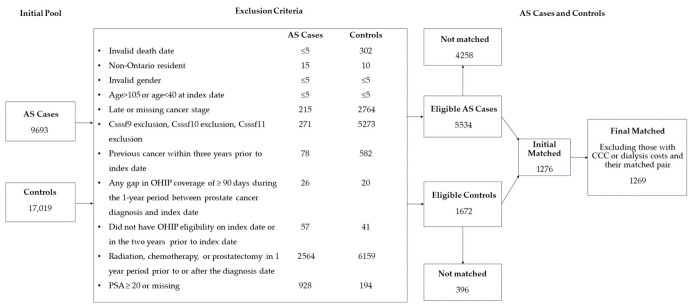
Study diagram for AS cases and non-AS cases. Abbreviations: CCC = Complex Continuing Care, CSSSF = CS Site-Specific Factor, OHIP = Ontario Health Insurance Plan. Note: To minimize risk of reidentification, ICES prohibits the presence of small cells (counts less than 5) in any output or report.

Individuals with erroneous dates (e.g., invalid death date, invalid birth date or invalid sex), non-Ontario residency, female gender, age of <40 or >105 years, and those diagnosed with PCa at stage III, IV or unknown/missing were excluded. A total of 1269 AS cases were matched to 1269 non-AS cases (Figure 1).

### 2.2. Data Sources

This study used healthcare administrative data located at the Institute for Clinical Evaluative Sciences (ICES) to determine HCRU and costs. The ICES Data Repository encompasses publicly funded administrative health services records for the Ontario population (~16 million people). In this study, various linked databases were utilized to gather information regarding cancer diagnosis, healthcare utilization and costing. These databases are for health services and include the following: the CIHI Continuing Care Reporting System, the CIHI Discharge Abstract Database, the CIHI National Ambulatory Care Reporting System, the CIHI National Rehabilitation Reporting System, CIHI Same Day Surgery, Ontario Health Insurance Plan (OHIP) billings, and the Ontario Home Care Database. Population data utilized the Registered Persons Database, while coding/geography data applied the Local Health Integration Network and Postal Code Conversion File. Other datasets utilized include the Ontario Case Costing Initiative, OCR, and the Ontario Laboratories Information System. Additional drug-specific databases are further described in the Outcome Variables sub-heading. A complete description of all the databases utilized in this study is found in Appendix A (Table A1).

### 2.3. Costing and Statistical Analyses

Healthcare costs were estimated using the GETCOST macro at ICES, a validated person-level costing methodology that aggregates costs across multiple healthcare sectors (e.g., hospitalizations, physician services, and outpatient care) in Ontario using administrative data and standardized costing algorithms. This approach assigns costs based on resource use and provincial costing data to generate comprehensive total healthcare expenditures for each individual [10]. In this study, costs were calculated as mean (standard deviation) and median (interquartile range) costs per patient-year (PPY), with total costs for each individual divided by their total follow-up time and standardized to 2023 Canadian dollars (CAD) to account for differences in follow-up duration. Further details and the validity of the GETCOST methodology have been described in previous studies [11,12,13]. All years were the timeframe used for the main analysis, in which AS cases and non-AS cases could have up to 6 years of follow-up. Statistical analyses were conducted at ICES and performed using SAS Enterprise Guide 8.3.

## 3. Results

### 3.1. Baseline Characteristics

Between 1 January 2010 and 31 December 2015, the baseline characteristics were determined for the low-risk prostate cancer cohort on AS and their matched non-AS cases. The majority of the baseline characteristics were well-matched between the two cohorts (Table 1).

### 3.2. Healthcare Resource Utilization and Costs

Table 2 presents HCRU results for AS cases and non-AS cases during the first actual year of AS (Year 0). Although the number of men who had cancer clinic visits was relatively low in both groups, though twice as high for non-AS cases versus AS cases (7.5% vs. 14.8%), the mean (1.7 vs. 26.7) and median (1.0 vs. 39.0) number of visits were significantly higher for non-AS cases. Meanwhile, specialist visits were highly utilized by both groups (99.6% for AS cases, 99.8% for non-AS cases), with AS cases having significantly lower mean number of visits (10.7 vs. 11.6). HCRU and cost results for All Years and Year 1 are in Appendix A Table A1, Table A2, Table A3, Table A4 and Table A5.

The mean and median costs (overall and per resource) for AS cases and non-AS cases in Year 0 are shown in Table 3. Compared to non-AS cases, AS cases had significantly lower mean overall costs ($6050 vs. $10,438, *p* < 0.0001), cancer clinic visit costs (mean: $489 vs. $26,290, *p* < 0.0001), and outpatient clinic visit costs (mean: $1586 vs. $2093, *p* < 0.0001). Median costs also showed significantly lower values for AS cases regarding cancer clinic visits ($368 vs. $30,609, *p* < 0.0001), outpatient clinic visits ($1299 vs. $1401, *p* = 0.03), all physician visits ($1163 vs. $1292, *p* = 0.01), and specialist visits ($983 vs. $1066, *p* = 0.01). Cost results for All Years and Year 1 are in Appendix A.

## 4. Discussion

This study evaluated the all-cause HCRU and mean overall costs for low-risk men diagnosed with PCa during their time on AS ($6100 ± $12,400) and for non-AS cases ($10,400 ± $17,800). During the first year after their PCa diagnosis (Year 0), the mean number of HCRU PPY was significantly lower for the AS cases versus controls in terms of cancer clinic visits, hospital outpatient clinic visits, all physician visits, and specialist visits. One possible explanation for the lower use of these resources is the AS protocol followed by AS patients during Year 0 and beyond, which may offer more consistent and structured healthcare encounters. In contrast, men in the control group may rely more heavily on clinic and physician visits to address both physical symptoms and psychological concerns. Thus, it is not surprising that the lower HCRU values for the AS cases are reflected in their lower mean overall cost PPY. Since costs trended lower for the AS cases over time, there is an economic benefit of a standardized AS program for low-risk men diagnosed with PCa.

The cost results associated with AS from this study can be contextualized by comparison to other PCa economic analyses with an AS comparator arm. A recent U.S. study evaluating real-world costs of first-line treatment versus AS reported a calculated cost per day of $2.97 for the AS cohort ($1084/year) and 22 healthcare encounters during a 5-year period [14], which is notably lower than the costs reported from our study. Sharma et al. (2019) investigated the cost-effectiveness of AS compared to various types of treatment in the US (e.g., radical prostatectomy (RP) and external beam radiotherapy (EBRT)) for patients with localized PCa [15]. Six years post-randomization, the mean per-patient cost was lowest for AS (US$12,143), followed by RP (US$17,781) and EBRT (US$29,238), which is higher than what our study found. While AS was considered a cost-effective approach for managing localized PCa in the initial years after diagnosis, the study emphasized that the relative cost-effectiveness of treatment emerges upon extended follow-up. Similarly, our study observed that the mean overall costs stabilized after the first year following the index date. An earlier Canadian analysis based on Quebec-specific costs estimated the mean first-year cost of AS to be approximately $6200 (2012 CAD) [9]. However, it is important to note that our study’s methodology differs from prior studies [9,15] that applied standardized unit reimbursement rates to model AS costs—often using Markov approaches, and that our study measured real-world healthcare utilization and costs based on actual AS and non-AS cases. This offers a more nuanced and empirically grounded understanding of the economic burden associated with AS in routine practice.

In Canada, groups such as Choosing Wisely Canada, Ontario Health and the Canadian Cancer Society recommend AS for very low- and low-risk PCa patients [16,17,18]. AS, over immediate treatment like RP, means that potential side effects such as erectile dysfunction, urinary incontinence, bleeding, and infection can be avoided [19]. Yet, anxiety, other mental health issues, and quality of life can influence the decision to continue with AS. McIntosh et al. (2022) surveyed and interviewed eligible participants (N = 166) about why they transitioned from AS to treatment [20], citing desire for action (51%), fear of progression (18%), and external pressure (15%). Medical factors included increased PSA (70%), physician recommendation (71%), biopsy results (63%), and Gleason score increase (60%). Despite these concerns, one systematic review showed that AS patients reported better than/similar to health-related quality of life (HRQOL) for patients who had radiation therapy or surgery, and comparable HRQOL for non-cancer patients [21]. While another systematic review reported similar HRQOL levels for AS patients, the authors highlighted uncertainty about AS follow-up protocols, and heterogeneity regarding the use of MRI and biomarkers [22] over invasive DRE and biopsies. It is worth noting that provinces like Alberta have put decision aids about choosing AS for PCa patients on their MyHealth. Alberta.ca website [23].

It is important to distinguish AS from watchful waiting (WW), another conservative management strategy typically recommended for men with limited life expectancy (e.g., less than 10 years). While AS involves structured monitoring via PSA tests, imaging, and biopsies, with the goal of intervening upon signs of progression, WW generally takes a more passive approach. In WW, treatment is only initiated when patients experience symptoms or clear signs of disease progression, and the intent is typically palliative rather than curative [24,25]. In contrast, AS is intended to preserve the option of curative treatment if disease progression occurs. Modelling studies have compared AS and WW in terms of life expectancy and quality of life [25,26,27], but also highlight the need for AS best practices to evolve. This could include the integration of newer diagnostic tools (e.g., MRI, genomic biomarkers), addressing informational gaps for patients and families, and providing psychological support tailored to individuals undergoing AS [27].

This study has several limitations. First, the sample of 1269 low-risk prostate cancer (PCa) patients on AS may not be fully representative of the broader PCa population, as it is skewed toward urban residents (88.2%) and individuals in the highest census-based income quintile (28.1%). Second, PSA values were measured only at baseline and used as a matching criterion for the non-AS cases, and tracking PSA changes over time could have provided insights into AS adherence and/or factored into the decision to seek treatment. It is important to note that PSA alone is not a reliable long-term marker for PCa treatment decisions [28]. Expanding diagnostic tools, such as additional biomarkers and assays, could further support AS by confirming a lower disease progression risk. During the study period (2010–2015), emerging biomarkers—including circulating tumour cells (CTCs) and genomic tests like Decipher and Prolaris—were increasingly integrated alongside PSA testing [29], although these tests are not currently funded under provincial healthcare plans. Third, survival outcomes were not collected for the 1269 AS cases and non-AS cases in this study that could be used with cost results and utility values to conduct a cost-effectiveness analysis of AS. One Canadian study by Klotz and colleagues (2015) reported 10- and 15-year actuarial cause-specific survival rates of 98.1% and 94.3%, respectively, for favourable- and intermediate-risk men on an AS protocol [8]. In addition, Timilshina et al. (2023) examined the long-term population-level oncological outcomes in low-grade PCa patients on AS and revealed a 10-year follow-up metastasis-free survival of 94.2%, overall survival of 88.7%, and cancer-specific survival of 98.1% [7]. However, long-term cancer-specific survival was slightly inferior with AS, showing a 1% reduction at 10 years, which must be balanced against the risks of overtreatment [7].

As mentioned previously, integrating genomic tests into clinical decision-making could enhance risk assessment prior/during/after AS, ultimately optimizing cost-effectiveness. While these tests are not yet widely reimbursed in Ontario, their broader adoption—similar to the Oncotype DX test in breast cancer—could refine treatment selection, improve outcomes, and reduce overall healthcare expenditures. The implementation of the Oncotype DX test was shown to theoretically reduce chemotherapy use in early-stage, hormone receptor-positive, HER2-negative, and node-negative breast cancer by 23%, translating to approximately $3.1 million CAD (2014) in cost savings [30]. The test provided value by identifying low-risk patients who could safely avoid chemotherapy and its associated toxicity while ensuring high-risk patients received appropriate treatment. Therefore, applying a similar precision medicine approach to AS could help better stratify men with low-risk prostate cancer, ensuring that men with low-risk PCa remain on surveillance while identifying men who may benefit from earlier intervention. The upfront cost of genomic testing for prostate cancer must be weighed against its potential to reduce downstream healthcare expenditures by preventing unnecessary interventions. As personalized medicine continues to evolve, incorporating genomic testing into AS protocols could improve patient outcomes, increase adherence, and enhance the overall cost-effectiveness of AS in prostate cancer management.

## 5. Conclusions

In conclusion, to our knowledge, this is the first Canadian study that provides real-world cost and resource utilization estimates associated with AS compared to non-AS cases. Unlike prior studies that relied on modelled cost estimates, our findings reflect actual HCRU and costs among patients on AS, reinforcing the economic value of a structured AS program for low-risk prostate cancer. However, a key challenge remains ensuring that men on AS do not transition to definitive treatment prematurely due to uncertainty or anxiety.

## Figures and Tables

**Table 1:** Baseline characteristics of matched AS and Non-AS Cases.

Characteristic	AS Cases *N* = 1269	Non-AS Cases *N* = 1269	Standard Deviation	*p*-Value
Age at Index Date				
Mean ± SD	65.58 ± 8.09	66.32 (7.81)	0.09	0.02
Median (IQR)	66 (60–71)	67 (61–71)	0.10	0.01
Rurality				
No (Urban)	1119 (88.2%)	1120 (88.3%)	0.00	0.95
Yes	150 (11.8%)	149 (11.7%)	0.00	
Income Quintile (*N* (%))				
1 (lowest)	195 (15.4%)	189 (14.9%)	0.01	0.94
2	220 (17.3%)	229 (18.0%)	0.02	
3	243 (19.1%)	237 (18.7%)	0.01	
4	254 (20.0%)	267 (21.0%)	0.03	
5 (highest)	357 (28.1%)	347 (27.3%)	0.02	
Charlson Index				
Mean ± SD	0.96 ± 1.20	0.96 ± 1.20	0.03	0.66
Median (IQR)	0 (0–2)	0 (0–2)	0.01	0.85
Charlson Index (Categorical) (*N* (%))				
0	334 (26.3%)	338 (26.6%)	0.01	0.75
1	51 (4.0%)	39 (3.1%)	0.05	
2	184 (14.5%)	189 (14.9%)	0.01	
3+	47 (3.7%)	52 (4.1%)	0.02	
No prior hospitalization—n (%)	653 (51.5%)	651 (51.3%)	0.00	
Collaborative Staging (*N* (%))				
Stage I	894 (70.4%)	846 (66.7%)	0.08	0.04
Stage II	375 (29.6%)	423 (33.3%)	0.08	
PSA				
Mean ± SD	6.78 ± 3.08	7.08 ± 3.13	0.10	0.02
Median (IQR)	6 (5–8)	7 (5–9)	0.12	0.00
PSA (categorical) (*N* (%))				
<10	1093 (86.1%)	1079 (85.0%)	0.03	0.43
10–20	176 (13.9%)	190 (15.0%)	0.03	

Note: PSA values for “missing” and “>20” were removed due to small cell values, which is why the PSA percentages do not add up to 100.

**Table 2:** Healthcare resource utilization for AS and non-AS Cases for Year 0.

Healthcare Resource	AS Cases Who Used Resource	Non-AS Cases Who Used Resource	Mean HCRU Use (SD)			Median HCRU Use (IQR)		
			AS Cases	Non-AS Cases	*p*-Value	AS Cases	Non-AS Cases	*p*-Value
Inpatient hospitalizations	100 (7.8%)	70 (5.5%)	1.3 (0.6)	1.2 (0.5)	0.71	1.0 (1.0–1.0)	1.0 (1.0–1.0)	0.78
Cancer clinic visits	95 (7.5%)	188 (14.8%)	1.7 (3.7)	26.7 (16.6)	<0.0001 *	1.0 (1.0–1.0)	39.0 (4.0–39.0)	<0.0001 *
Outpatient clinic visits	947 (74.6%)	1032 (81.3%)	3.6 (2.7)	4.9 (4.1)	<0.0001 *	3.0 (2.0–5.0)	3.0 (2.0–6.0)	<0.0001 *
Homecare visits	57 (4.5%)	62 (4.9%)	23.6 (42.9)	17.7 (40.9)	0.44	9.0 (3.0–25.0)	5.0 (2.0–10.0)	0.03 *
All physician visits	1267 (99.8%)	1268 (99.9%)	16.3 (13.2)	17.5 (13.0)	0.02 *	13.0 (8.0–20.0)	14.0 (9.0–22.0)	0.001 *
General Practitioner visits	1020 (80.4%)	1025 (80.8%)	5.0 (5.4)	5.0 (5.4)	0.91	3.0 (2.0–6.0)	3.0 (2.0–6.0)	0.65
All specialist visits	1264 (99.6%)	1267 (99.8%)	10.7 (10.2)	11.6 (9.5)	0.02	8.0 (5.0–13.0)	9.0 (6.0–15.0)	0.0005 *

Note: All costs have been rounded to the nearest $100 to reflect appropriate precision given the variability in the data. * indicates *p* ≤ 0.05.

**Table 3:** Costs for AS and non-AS Cases for Year 0.

Resource	Mean Cost per Person-Year (SD)			Median Cost per Person-Year (IQR)		
	AS Cases	Non-AS Cases	*p*-Value	AS Cases	Non-AS Cases	*p*-Value
Overall costs	$6100 ($12,400)	$10,400 ($17,800)	<0.0001 *	$3500 ($2100–$5700)	$3700 ($2100–$7200)	0.0001 *
Inpatient hospitalizations	$13,900 ($20,300	$16,100 ($28,900)	0.56	$7600 ($4600–$16,500)	$9900 ($5800–$15,700)	0.16
Cancer clinic visits	$500 ($600)	$26,300 ($18,100)	<0.0001 *	$400 ($300–$500)	$30,600 ($2100–$45,100)	<0.0001 *
Outpatient clinic visits	$1600 ($1200)	$2100 ($1900)	<0.0001 *	$1300 ($900–$2100)	$1400 ($800–$2500)	0.03 *
Homecare visits	$2900 ($3200)	$2100 ($2400)	0.14	$1600 ($1100–$3200)	$1300 ($900–$2000)	0.03 *
All physician visits	$1900 ($2400)	$2000 ($2200)	0.14	$1200 ($700–$2200)	$1300 ($700–$2600)	0.01 *
GP visits	$300 ($500)	$300 ($900)	0.89	$200 ($0–$400)	$200 ($100–$400)	0.54
All specialist visits	$1500 ($2100)	$1700 ($1800)	0.15	$1000 ($500–$1900)	$1100 ($500–$2300)	0.01 *

Note: All costs have been rounded to the nearest $100 to reflect appropriate precision given the variability in the data. * indicates *p* ≤ 0.05.

## Data Availability

The dataset from this study is held securely in coded form at ICES. While legal data sharing agreements between ICES and data providers (e.g., healthcare organizations and government) prohibit ICES from making the dataset publicly available, access may be granted to those who meet pre-specified criteria for confidential access, available at www.ices.on.ca/DAS (email: das@ices.on.ca). The full dataset creation plan and underlying analytic code are available from the authors upon request, understanding that the computer programs may rely upon coding templates or macros that are unique to ICES and are therefore either inaccessible or may require modification.

## Abbreviations

The following abbreviations are used in this manuscript:

HCRU	Healthcare Resource Utilization
PCa	Prostate Cancer
AS	Active Surveillance
PSA	Prostate-Specific Antigen
DRE	Digital Rectal Exam
GG	Grade Group
GS	Gleason Score
TRUS	Transrectal Ultrasound
CCO	Cancer Care Ontario
CAD	Canadian Dollars
PPY	Per Patient-Year
OHIP	Ontario Health Insurance Plan
OCR	Ontario Cancer Registry
CCI	Charlson Comorbidity Index
LHIN	Local Health Integration Network
CIHI	Canadian Institute for Health Information
ICES	Institute for Clinical Evaluative Sciences
SD	Standard Deviation
IQR	Interquartile Range
GP	General Practitioner
HRQOL	Health-Related Quality of Life
MRI	Magnetic Resonance Imaging
CTC	Circulating Tumour Cell
RP	Radical Prostatectomy
EBRT	External Beam Radiotherapy
WW	Watchful Waiting

## Appendix A

**Table A1:** ICES data sources.

Discharge Abstract Database (DAD)
The DAD is compiled by the Canadian Institute for Health Information and contains administrative, clinical (diagnoses and procedures/interventions), demographic, and administrative information for all admissions to acute care hospitals, rehab, chronic, and day surgery institutions in Ontario. At ICES, consecutive DAD records are linked together to form ‘episodes of care’ among the hospitals to which patients have been transferred after their initial admission.
National Ambulatory Care Reporting System (NACRS)
The NACRS is compiled by the Canadian Institute for Health Information and contains administrative, clinical (diagnoses and procedures), demographic, and administrative information for all patient visits made to hospital- and community-based ambulatory care centres (emergency departments, day surgery units, haemodialysis units, and cancer care clinics). At ICES, NACRS records are linked with other data sources (DAD, OMHRS) to identify transitions to other care settings, such as inpatient acute care or psychiatric care.
Ontario Health Insurance Plan Claims Database (OHIP)
The OHIP claims database contains information on inpatient and outpatient services provided to Ontario residents eligible for the province’s publicly funded health insurance system by fee-for-service healthcare practitioners (primarily physicians) and “shadow billings” for those paid through non-fee-for-service payment plans. The main data elements include patient and physician identifiers (encrypted), code for service provided, date of service, associated diagnosis, and fee paid.
Registered Persons Database (RPDB) files
The RPDB provides basic demographic information (age, sex, location of residence, date of birth, and date of death for deceased individuals) for those issued an Ontario health insurance number. The RPDB also indicates the time periods for which an individual was eligible to receive publicly funded health insurance benefits and the best-known postal code for each registrant on July 1st of each year.
Same Day Surgery Database (SDS)
The SDS is compiled by the Canadian Institute for Health Information and contains administrative, clinical (diagnoses and procedures), demographic, and administrative information for all patient visits made to day surgery institutions in Ontario. The main data elements include patient demographics, clinical data (diagnoses, procedures, physician), administrative data (institution/hospital number, etc.), financial data, service-specific data elements for day surgery and emergency departments.
Ontario Cancer Registry (OCR)
The OCR is collected by Cancer Care Ontario and contains information on all Ontario residents who have been newly diagnosed with cancer (“incidence”) or who have died of cancer (“mortality”). All new cases of cancer are registered, except non-melanoma skin cancer.
Ontario Drug Benefit Claims (ODB)
The ODB database contains prescription medication claims for those covered under the provincial drug program, mainly: those aged 65 years and older, nursing home residents, patients receiving services under the Ontario Home Care program, those receiving social assistance, and residents eligible for specialized drug programs. Main data elements include drug identifier, quantity, number of days supplied, date dispensed, cost, and patient, pharmacy, and physician identifiers.
National Rehabilitation Reporting System (NRS)
The NRS is compiled by the Canadian Institute for Health Information and contains client data collected from participating adult inpatient rehabilitation facilities and programs across Canada. Main data elements contain socio-demographic information, administrative data (e.g., referral, admission, and discharge), health characteristics, activities, and participation (e.g., ADL, communication, social interaction), and interventions. (Note—only available up to MAR 2022)
Postal Code Conversion File (PCCF)
The PCCF database will link to postal codes within a given cohort and determine other census geographic identifiers, such as dissemination/enumeration area, census division, longitude/latitude, urban/rural flag, and neighbourhood income quintile.
Home Care Database (HCD)
The Home Care Database is maintained by Health Shared Services Ontario and is a clinical, client-centred database that captures all home care services provided or coordinated by Local Health Integration Networks. This dataset contains information on client, intake, assessment, admission, diagnostic and surgical procedures, and service delivery.
New Drug Funding Program (NDFP)
The NDFP database is collected by Cancer Care Ontario and captures the use of new, often expensive, cancer drugs. The program was created in 1995 to ensure that Ontario patients have equal access to high-quality intravenous (IV) cancer drugs. The data includes a list of drugs, frequency by drug name, patient, and treatment data, including size (height, weight) and dosage.
Activity Level Reporting (ALR)
The ALR database is collected by Cancer Care Ontario and represents the basic set of data elements required to produce the quality, cost, and performance indicators for the cancer system. The data elements constitute patient-level activity within the cancer system focused on radiation and systemic therapy services and outpatient oncology clinic visits. This data is also a key component of the Ontario Cancer Registry (OCR), which registers every malignant neoplasm diagnosed in Ontario.

**Table A2:** Healthcare resource utilization for AS and non-AS cases for All Years.

Healthcare Resource Utilization	AS Cases Who Used Resource	Non-AS Cases Who Used Resource	Mean HCRU Use (SD)			Median HCRU Use (IQR)		
			AS Cases	Non-AS Cases	*p*-Value	AS Cases	Non-AS Cases	*p*-Value
Inpatient hospitalizations	540 (42.6%)	537 (42.3%)	0.3 (0.3)	0.4 (0.5)	0.01 *	0.2 (0.2–0.3)	0.2 (0.2–0.5)	0.08
Cancer clinic visits	286 (22.5%)	313 (24.7%)	4.6 (5.4)	4.2 (4.3)	0.25	3.9 (1.0–6.5)	3.5 (1.2–6.0)	0.15
Outpatient clinic visits	1146 (90.3%)	1167 (92.0%)	2.3 (2.1)	2.7 (2.8)	0.0005 *	2.0 (0.8–3.0)	2.0 (1.0–3.3)	0.02 *
Homecare visits	316 (24.9%)	323 (25.5%)	15.4 (113.0)	16.0 (58.0)	0.92	1.2 (0.5–3.2)	1.5 (0.7–5.7)	0.04 *
All physician visits	1260 (99.3%)	1265 (99.7%)	15.0 (11.9)	16.7 (14.0)	0.0009 *	11.8 (8.0–18.1)	13.2 (9.0–19.8)	<0.0001 *
GP visits	1210 (95.4%)	1233 (97.2%)	4.0 (5.7)	4.3 (5.3)	0.19	2.4 (1.2–4.8)	2.7 (1.3–5.5)	0.008 *
All specialist visits	1253 (98.7%)	1262 (99.5%)	9.4 (8.3)	10.4 (9.0)	0.008 *	7.3 (4.8–11.0)	7.9 (5.2–12.7)	0.0009 *

Note: * indicates *p* ≤ 0.05.

**Table A3:** Healthcare resource utilization for AS and non-AS Cases for Year 1.

Healthcare Resource Utilization	AS Cases Who Used Resource	Non-AS Cases Who Used Resource	Mean HCRU Use (SD)			Median HCRU Use (IQR)		
			AS Cases	Non-AS Cases	*p*-Value	AS Cases	Non-AS Cases	*p*-Value
Inpatient hospitalizations	190 (15.0%)	169 (13.3%)	1.2 (0.5)	1.3 (0.7)	0.19	1.0 (1.0–1.0)	1.0 (1.0–1.0)	0.64
Cancer clinic visits	119 (9.4%)	129 (10.2%)	18.1 (17.4)	16.3 (14.3)	0.38	11.0 (2.0–39.0)	16.0 (2.0–23.0)	0.90
Outpatient clinic visits	879 (69.3%)	934 (73.6%)	3.7 (3.1)	4.1 (3.4)	0.01 *	3.0 (2.0–5.0)	3.0 (2.0–5.0)	0.01 *
Homecare visits	94 (7.4%)	105 (8.3%)	37.8 (199.3)	28.1 (85.2)	0.65	5.0 (3.0–11.0)	6.0 (3.0–15.0)	0.11
All physician visits	1250 (98.5%)	1258 (99.1%)	15.8 (14.2)	17.3 (14.9)	0.01 *	12.0 (7.0–20.0)	13.3 (8.0–21.0)	0.0007 *
GP visits	960 (75.7%)	1022 (80.5%)	5.1 (6.6)	5.0 (5.8)	0.79	3.0 (2.0–6.0)	3.0 (2.0–6.0)	0.84
All specialist visits	1218 (96.0%)	1246 (98.2%)	10.6 (10.6)	11.3 (10.7)	0.10	7.0 (4.0–14.0)	8.0 (4.0–15.0)	0.03 *

Note: * indicates *p* ≤ 0.05.

**Table A4:** Costs for AS and non-AS Cases for All Years.

Resource	Mean Cost per Person-Year (SD)			Median Cost per Person-Year (IQR)		
	AS Cases	Non-AS Cases	*p*-Value	AS Cases	Non-AS Cases	*p*-Value
Overall costs	$7400 ($14,000)	$8500 ($14,700)	0.06	$4400 ($2300–$8300)	$4800 ($2500–$9500)	0.008 *
Inpatient hospitalizations	$3600 ($8500)	$4200 ($12,700)	0.31	$1600 ($1400–$3300)	$1800 ($1400–$3700)	0.18
Cancer clinic visits	$2800 ($3700)	$3000 ($5800)	0.62	$2200 ($5700–$3800)	$1700 ($600–$3200)	0.20
Outpatient clinic visits	$1000 ($900)	$1100 ($1200)	0.0003 *	$800 ($400–$1200)	$800 ($400–$1400)	0.008 *
Homecare visits	$1900 ($17,800)	$1300 ($3100)	0.57	$300 ($200–$600)	$300 ($200–$900)	0.02 *
All physician visits	$1700 ($1800)	$1900 ($1900)	0.02 *	$1200 ($800–$2100)	$1400 ($800–$2300)	0.003 *
GP visits	$200 ($500)	$200 ($400)	0.87	$100 ($0–$300)	$100 ($0–$300)	0.03 *
All specialist visits	$1400 ($1500)	$1600 ($1600)	0.07	$1000 ($600–$1800)	$1200 ($600–$1900)	0.01 *

Note: * indicates *p* ≤ 0.05.

**Table A5:** Costs for AS and non-AS cases for Year 1.

Resource	Mean Cost per Person-Year (SD)			Median Cost per Person-Year (IQR)		
	AS Cases	Non-AS Cases	*p*-Value	AS Cases	Non-AS Cases	*p*-Value
Overall costs	$8100 ($16,300)	$8500 ($15,100)	0.51	$3100 ($1700–$8400)	$3700 ($1800–$9400)	0.008 *
Inpatient hospitalizations	$11,100 ($18,000)	$12,800 ($21,100)	0.41	$8700 ($8300–$9900)	$8500 ($8100–$11,900)	0.98
Cancer clinic visits	$12,900 ($14,200)	$10,600 ($12,400)	0.18	$5800 ($1300–$23,500)	$7500 ($1600–$11,700)	0.61
Outpatient clinic visits	$1500 ($1300)	$1700 ($1400)	0.04 *	$1200 ($800–$2000)	$1200 ($800–$2200)	0.62
Homecare visits	$5600 ($32,300)	$2600 ($3800)	0.35	$1300 ($1100–$2200)	$1400 ($1100–$2300)	0.70
All physician visits	$2000 ($2500)	$2100 ($2400)	0.54	$1100 ($500–$2500)	$1300 ($600–$2700)	0.04 *
GP visits	$300 ($600)	$300 ($400)	0.19	$200 ($100–$400)	$200 ($100–$400)	0.90
All specialist visits	$1800 ($2300)	$1800 ($2200)	0.91	$900 ($400–$2100)	$1000 ($400–$2400)	0.25

Note: * indicates *p* ≤ 0.05.
